# Methylation in the promoter regions of *WT1, NKX6-1 and DBC1* genes in cervical cancer tissues of Uygur women in Xinjiang

**DOI:** 10.1590/1678-4685-GMB-2016-0146

**Published:** 2018

**Authors:** Dan Wu, Jinli Zhang, Peiwen Fan, Hongtao Li, Dongmei Li, Huan Pan, Hongchang He, Xianxian Ren, Zhenzhen Pan, Renfu Shao, Zemin Pan

**Affiliations:** 1 Department of Biochemistry and Molecular Biology, School of Medicine, Shihezi University, Xinjiang Endemic and Ethnic Disease and Education Ministry Key Laboratory, Shihezi, Xinjiang, China; 2 Genecology Research Centre, School of Science and Engineering, University of the Sunshine Coast, Maroochydore DC, Queensland, Australia; 3 Clinical Laboratory, Branch of the First Affiliated Hospital of Xinjiang Medical University, Changji, Xinjiang, China

**Keywords:** gene methylation, gene expression, HPV16/18, cervical cancer, Uygur women

## Abstract

This study aimed to explore: 1) DNA methylation in the promoter regions of Wilms tumor gene 1 (*WT1*), NK6 transcription factor related locus 1 gene (*NKX6-1*) and Deleted in bladder cancer 1 (*DBC1*) gene in cervical cancer tissues of Uygur women in Xinjiang, and 2) the correlation of gene methylation with the infection of HPV16/18 viruses. We detected HPV16/18 infection in 43 normal cervical tissues, 30 cervical intraepithelial neoplasia lesions (CIN) and 48 cervical cancer tissues with polymerase chain reaction (PCR) method. Methylation in the promoter regions of the *WT1, NKX6-1* and *DBC1* genes in the above-mentioned tissues was measured by methylation-specific PCR (MSP) and cloning sequencing. The expression level of these three genes was measured by real-time PCR (qPCR) in 10 methylation-positive cervical cancer tissues and 10 methylation-negative normal cervical tissues. We found that the infection of HPV16 in normal cervical tissues, CIN and cervical cancer tissues was 14.0, 36.7 and 66.7%, respectively. The infection of HPV18 was 0, 6.7 and 10.4%, respectively. The methylation rates of *WT1, NKX6-1* and *DBC1* genes were 7.0, 11.6 and 23.3% in normal cervical tissues, 36.7, 46.7 and 30.0% in CIN tissues, and 89.6, 77.1 and 85.4% in cervical cancer tissues. Furthermore, *WT1, NKX6-1* and *DBC1* genes were hypermethylated in the high-grade squamous intraepithelial lesion (CIN2, CIN3) and in the cervical cancer tissues with infection of HPV16/18 (both *P*< 0.05). The expression of *WT1, NKX6-1* and *DBC1* was significantly lower in the methylation-positive cervical cancer tissues than in methylation-negative normal cervical tissues. Our findings indicated that methylation in the promoter regions of *WT1, NKX6-1* and *DBC1* is correlated with cervical cancer tumorigenesis in Uygur women. The infection of HPV16/18 might be correlated with methylation in these genes. Gene inactivation caused by methylation might be related to the incidence and development of cervical cancer.

## Introduction

Cervical cancer is a common gynecologic malignancy with its incidence and mortality ranked third and fourth, respectively, in women malignant tumors (Ferlay *et al.*, 2013). A third of the world’s morbidity and mortality from cervical cancer is in China (Gao *et al.*, 2007). Xinjiang is a high-incidence region of cervical cancer in China, especially in its southern part. The incidence and mortality of cervical cancer are higher in Uygur than in Han women and other ethnic groups who live in the same region. Therefore, cervical cancer is a major threat to Uygur women’s health in Xinjiang (Pan *et al.*, 2010). Human papillomavirus (HPV) infection is one of the most important factors related to cervical cancer (Huang *et al.*, 2012) and its persistency is a prerequisite for cervical cancer and its precursor lesions (Moscicki *et al.*, 2008; Dempsey and Mendez, 2010). It has been suggested that epigenetic changes can also cause cervical cancer (Sova *et al.*, 2006).

Development and progression of cervical cancer is caused by a combination of virus, proto-oncogenes, tumor suppressor genes and immune factors. In developing countries, due to poor early diagnosis, precancerous lesions are not found in time to receive the best treatment, making the mortality of cervical cancer far higher than in developed countries (Parkin *et al.*, 2000). Additionally to DNA sequence changes (i.e. mutations and deletions), DNA methylation is suggested as a mechanism for cervical cancer by inactivating tumor suppressor genes (Buysschaert *et al.*, 2008). Epigenetic changes can regulate gene expression and DNA methylation is an important component of the epigenetic modifications that cause cancer (Feinberg and Tycko, 2004). Previous studies have found that high methylation can cause suppressor gene inactivation in cancer tissues. The *WT1, NKX6-1* and *DBC1* genes in malignant tumor tissues are prone to high methylation (Grønbæk *et al.*, 2008, Bruno *et al.*, 2012, Shimazu *et al.*, 2015). Thus, gene methylation analysis combined with HPV infection detection can be used in the early diagnosis of cervical cancer.

The *WT1* gene was first identified in kidney tumor on human chromosome 11p13. *WT1* comprises ~5 kb and contains 10 exons; its mRNA spans ~2.9 kb, coding for the renal tumor protein (Wilms tumor protein), which has 449 amino acids (Breslow *et al.*, 1993). Breslow *et al.* (1993) found that WT1 protein is a transcriptional regulation factor. It can activate or inhibit the expression of target genes, producing different biological effects. WT1 plays a role in regulating cell proliferation, growth, differentiation and apoptosis (Scharnhorst *et al.*, 2001) and can be both a tumor suppressor and a carcinogenic inducer. Moreover, *WT1* has been found hypermethylated in many tumors including glioblastoma, prostate cancer and ovarian cancer (Jacobs *et al.*, 2013; Jiang *et al.*, 2014; Rankeillor *et al.*, 2014).

The *NKX6-1* gene is located in human chromosome 4q21.2-q22, its coding region comprises ~4.9 kb with three exons. This gene codes for a protein of 367 amino acids (Inoue *et al.*, 1997). *NKX6-1*, which was identified initially in rodents, is a specific transcription factor for islet beta cells and is crucial for their differentiation in the pancreas.

The *DBC1* gene is located in human chromosome 9q32-33 (Habuchi *et al.*, 1998); The DBC1 protein is a member of the RHO atypical family, which contains small GTP enzymes. *DBC1* loses heterozygosity in many cancers and is a new gene with hypermethylation status in malignant tumor tissues. It has been shown that DBC1 gene expression increases cell death in bladder cancer cell line (Wright *et al.*, 2004) and inhibits the growth of non-small cell lung cancer (Izumi *et al.*, 2005).

We investigated the relationship between gene methylation and infection of HPV16 and HPV18 in cervical cancer. We aimed to understand the expression of *WT1, NKX6-1* and *DBC1* in the cervical cancer of Uygur women in Xinjiang, and the potential of methylation markers for the screening of cervical cancer.

## Materials and Methods

### Sample collection

Forty-three normal cervical tissues, 30 cervical intraepithelial neoplasia lesions (CIN) and 48 cervical cancer tissues were collected at the First and Third Affiliated Hospital, School of Medicine, Shihezi University, and the First People’s Hospital of Kashgar. All samples were fresh biopsy tissues from Uygur women who had no radiation nor chemotherapy treatment. All samples were examined by at least two pathologists. Ethical approval for this study was granted by the hospitals with informed consent from patients and their families. The samples were stored in -80 °C freezer.

### DNA extraction and HPV detection

Genomic DNA was extracted with TIANamp FFPE DNA Kit DP331-02 Kit (TIANGEN, Beijing), checked by agarose gel (0.7%) electrophoresis for quality and stored at -20 °C. All samples were assessed for high-risk HPV16/18 by PCR with specific primers (Table 1).

**Table 1:** Primer sequences for PCR analysis.

Gene Name	Primer Sequence (5’-3’)	Product Size(bp)	Annealing Temperature(°C)
HPV16	F:5’-GACCCAGAAAGTTACCACAG-3’	268	57
	R:5’-CACAACGGTTTGTTGTATTG-3’		
HPV18	F:5’-TGCCAGAAACCGTTGAATCC-3’	268	55
	R:5’-TCTGAGTCGCTTAATTGCTC-3’		
WT1QX (M)	F:5’-TGTTGAGTGAATGGAGCGGTC-3’	147	59
	R:5’-CGAAAAACCCCCGAATATAAACG-3’		
WT1QX (U)	F:5’-TGTTGAGTGAATGGAGTGGTT-3’	151	59
	R:5’-AATTACAAAAAACCCCCAAATATAAACAC-3’		
WT1HY (M)	F:5’-GTTAGGCGTCGTCGAGGTTA-3’	206	60
	R:5’-AAAACGCAAAATCCAACACC-3’		
WT1HY (U)	F:5’-TGGGATTTGGGTGGTATTTG-3’	216	60
	R:5’-CACCAACACCCACTACACCA-3’		
NKX6-1 (M)	F:5’-CGTGGTCGTGGGATGTTAGC-3’	146	60
	R:5’-ACAAACAACGAAAAATACGCG-3’		
NKX6-1 (U)	F:5’-TGTGGTTGTGGGATGTTAGT-3’	148	60
	R:5’-CAACAAACAACGAAAAATACGCGA-3’		
DBC1(M)	F:5’-TTGTAAATTGATTTGGCGCGC-3’	253	59
	R:5’-TTCCGAACACGACGCGAAA-3’		
DBC1(U)	F:5’-TTTATGGTTGTAAATTGATTTGGTGTGT-3’	269	59
	R:5’-CAACTCACATTCCAAACACAACACA-3’		
β-actin-qRT	F:5’-CCCAGCACAATGAAGATCAAGATCAT-3’	101	56
	R:5’-ATCTGCTGGAAGGTGGACAGCG -3’		
WT1-qRT	F:5’-ACTCTTGTACGGTCGGCATC-3’	127	55
	R:5’-TCTCACCAGTGTGCTTCCTG-3’		
NKX6-1-qRT	F:5’-CCAACACGAGACCCACTTTT-3’	122	55
	R:5’-CTCTGTCATCCCCAACGAAT-3’		
DBC1-qRT	F:5’-TCCTGTTTATATGGGGCCGTA-3’	171	56
	R:5’-TGGTTGTAAATCCTTGACGGTG-3’		

M: methylated-specific primer; U: unmethylated-specific primer; F: forward primer; R: reverse primer

### Bisulfite conversion and methylation-specific PCR (MSP)

The genomic DNA (1 μg) was bisulfite-modified using CpGenomeTM DNA Modification Kit (S7820, CHEMICON, American) according to the manufacturer’s recommendations and dissolved in 30 μL of nuclease-free water. The methylation and non-methylation primers and their optimal annealing temperatures for *WT1, NKX6-1* and *DBC1* are listed in Table 1. *In vitro* methylated DNA (IVD) was used as the positive control.

### Cloning sequencing of MSP products

Four microliters of PCR product was used to link with T vector by pEASY-T1 Cloning kit (TransGen Biotech, Beijing) according to the manufacturer’s instruction. *E. coli* DH5α competent cells and LB agar plates coated with ampicillin (AMP), IPTG and X-gal were used in the transformation. Colonies were grown at 37 °C for 12-16 h. Positive white colonies for methylated and unmethylated *WT1, NKX6-1* and *DBC1* genes were selected and the plasmids were extracted. PCR further confirmed the colonies, and gene sequence analysis confirmed the MSP of the gene fragments.

### RNA extraction and RT-qPCR

Total RNA was prepared with Trizol (Invitrogen) following the manufacturer’s instruction. cDNA was produced from 1 μg of RNA using the RevertAid First Strand cDNA Synthesis Kit (K1622, Thermo, American). Gene expression was analyzed by real-time PCR (qPCR) with the QuantiFast SYBR Green PCR Kit (QIAGEN). The primers used are listed in Table 1. β-actin was used as the internal control.

### Statistical analysis

SPSS 17.0 software was used for statistical analysis. Methylation in the promoter regions of *WT1, NKX6-1* and *DBC1* was analyzed with chi-square test. The respective mRNA levels in cervical cancer tissues and normal cervical tissues were analyzed by Student’s t-test. *P*< 0.05 was considered statistically significant.

## Results

### Infection of HPV16/18 in cervical tissues

We found that six of the 43 normal cervical tissues, 11 of the 30 CIN lesions and 32 of the 48 cervical cancer tissues were infected with HPV16. HPV18 infection was not found in normal cervical tissues but was found in two of the 30 CIN lesions and five of the 48 cervical cancer tissues. The positive cases of HPV16 infection in CIN1, CIN2, CIN3 were 1, 4 and 6, respectively. The positive cases of HPV18 infection in the above tissues were 0, 1, 1 (Table 2). There was only one tumor sample exclusively positive for HPV18 infection; the other four HPV18 positive samples were also co-infected by HPV16 (Table 3). The difference in HPV16 infection rate among normal, CIN and cervical cancer tissues was statistically significant (*P*< 0.01). However, the difference in HPV18 infection rate among those tissues was not statistically significant.

**Table 2:** Infection status of HPV16/18 in CIN tissues.

	HPV16			HPV18		
Group	Infection Ratio (%)	χ^2^	*P*	Infection Ratio (%)	χ^2^	*P*
CIN1	10.0(1/10)	1.067	0.302	0.0(0/10)	0.0	1.000
CIN2	40.0(4/10)	0.200	0.655	10.0(1/10)	0.0	1.000
CIN3	60.0(6/10)	3.516	0.061	10.0(1/10)	0.0	1.000

**Table 3:** Infection status of HPV16/18.

	HPV16			HPV18		
Group	Infection Ratio (%)	χ^2^	*P*	Infection Ratio (%)	χ^2^	*P*
Normal	14.0(6/43)	36.815	0.000^*^	0.0(0/43)	0.976	0.323
CIN	36.7(11/30)	6.717	0.010^Δ^	6.7(2/30)	0.025	0.876
Cancer	66.7(32/48)	25.914	0.000^#^	10.4(5/48)	2.946	0.086

* Normal group compared with CIN group (*P*< 0.05)

Δ CIN group compared with Cancer group (*P*< 0.05)

# Normal group compared with Cancer group (*P*< 0.05)

### Methylation of *WT1, NKX6-1* and *DBC1*

**Figure 1:**
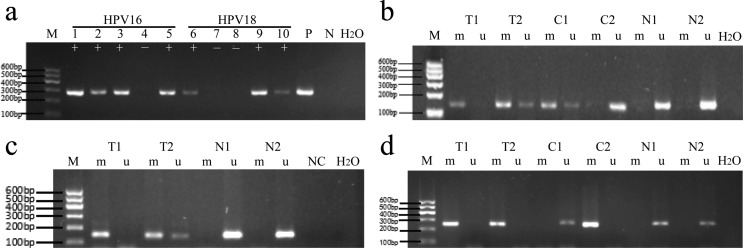
Infection with high-risk human papillomavirus (hr-HPV) and methylation of WT1, NKX6-1 and DBC1 genes in different stages of cervical lesions by agarose gel electrophoresis. (A) HPV16/18 infection; (B) WT1 methylation; (C) NKX6-1 methylation; (D) DBC1 methylation. M: marker (100 ~ 600 bp); lanes 1-5: HPV16 virus PCR products; lanes 6-10: HPV18 virus PCR products; P: positive control, N: negative control; +: positive, -: negative. M: methylation-specific PCR products; U: unmethylation-specific PCR products; T: cervical cancer tissue; C: cervical intraepithelial neoplasia lesions; N: normal cervical tissue.

The methylation rate of *WT1* in normal cervical tissues, CIN tissues and cervical cancer tissues (Tables 4 and 5) was 7.0, 36.7 and 89.6%, respectively. The methylation rate of NKX6-1 gene in these tissues was 11.6, 46.7 and 77.1%. The methylation rate of DBC1 gene in these tissues was 23.3%, 30% and 85.4%. The corresponding results of agarose gel electrophoresis are shown in Figure 1.

**Table 4:** Methylation ratio of *WT1, NKX6-1, DBC1.*

	Methylation Ratio (%)				
Gene Name	Normal	CIN	Cancer	χ^*2*^	*P*
WT1	7.0	36.7	89.6	63.863	0.000*
NKX6-1	11.6	46.7	77.1	39.089	0.000*
DBC1	23.3	30.0	85.4	41.180	0.000*

Note: using chi-square test, *P*< 0.05

**Table 5:** Methylation ratios of *WT1, NKX6-1, DBC1* in CIN tissues.

	WT1			NKX6-1			DBC1		
Group	Methylation Ratio (%)	χ^2^	*P*	Methylation Ratio (%)	χ^2^	*P*	Methylation Ratio (%)	χ^2^	*P*
CIN1	0.0 (0/10)	1.569	0.211	30.0 (3/10)	0.000	1.000	10.0 (1/10)	0.000	1.000
CIN2	30.0 (3/10)	3.232	0.070	40.0 (4/10)	0.808	0.370	20.0 (2/10)	1.875	0.170
CIN3	80.0 (8/10)	10.208	0.001^#^	70.0 (7/10)	1.800	0.179	60.0 (6/10)	3.516	0.057

# CIN1 group compared with CIN3 group (*P*< 0.05)

**Figure 2:**
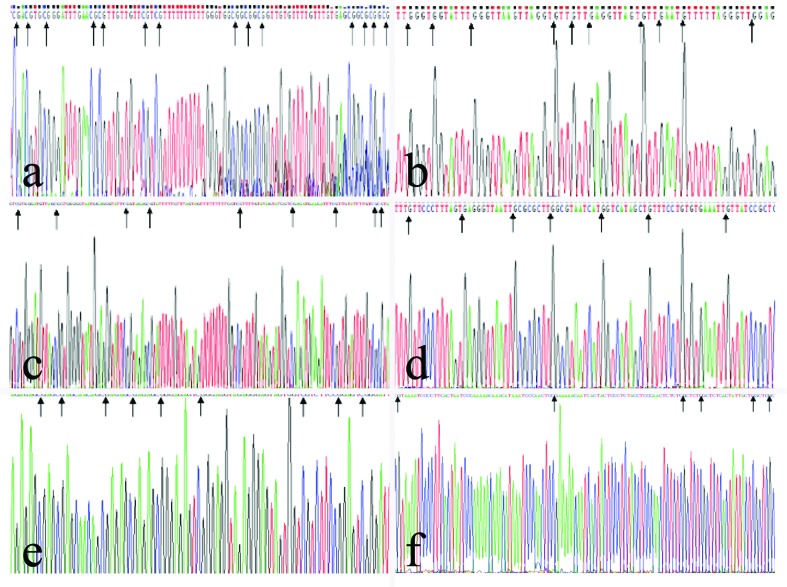
Sequencing of MSP products. Methylated C in CpG loci remained unchanged whereas unmethylated C residues were modified into T (partial modifications do not change into T). A, B: methylation in the promoter region of the *WT1* gene; C, D: methylation in the promoter region of the *NKX6-1* gene; E, F: methylation in the promoter region of the *DBC1* gene. Left lane: methylation products; right lane: unmethylated products; arrows indicate CpG loci.

Cloning and sequencing of the MSP products showed that after bisulfite modification the methylated CpG in C sites did not change, whereas the unmethylated C sites changed to the base of T (Figure 2). We analyzed statistically the relationship of the methylation rates of *WT1, NKX6-1* and *DBC1* with patient age and the staging of the International Federation of Gynecology and Obstetrics (FIGO), in 48 cervical cancer tissues; there was no statistically significant difference (Table 6).

**Table 6:** Correlation of promoter region methylation with clinical factors of cervical cancer patients.

		WTI			NKX6-1			DBC1		
Clinical Factors	Total	Methylation Ratio (%)	χ^2^	*P*	Methylation Ratio (%)	χ^2^	*P*	Methylation Ratio (%)	χ^2^	*P*
Age										
<50	25	92.0 (23/25)	0.010	0.922	72.0 (18/25)	0.763	0.382	80.0 (20/25)	0.489	0.484
≥50	23	87.0 (20/23)			82.6 (19/23)			91.3 (21/23)		
FIGO staging										
I	33	87.9 (29/33)			91.0 (30/33)			81.8 (27/33)		
II	10	90.0 (9/10)	2.733	0.218	90.0 (9/10)	1.204	0.761	70.0 (7/10)	1.921	0.366
III	5	60.0 (3/5)			80.0 (4/5)			60.0 (3/5)		

### Correlation between the methylation status of *WT1, NKX6-1* and *DBC1* and HPV16/18 infection

In the 20 high-grade squamous intraepithelial lesions (CIN2, CIN3) and the 48 cervical cancer tissue samples, the methylation rates of *WT1* and *DBC1* in the HPV16/18 positive group were significantly higher than those in the HPV16/18 negative group (*P*< 0.05). The methylation of *NKX6-1*, however, showed no significant difference between the two groups (Table 7).

**Table 7:** Promoter of gene methylation and HPV16/18 infection distribution in CIN2, CIN3 and cervical cancer tissue.

		WT1		NKX6-1		DBC1	
Group	Total	Methylation	Unmethylation	Methylation	Unmethylation	Methylation	Unmethylation
HPV16/18 positive	42	37	5	32	10	34	8
HPV16/18 negative	26	17	9	18	8	14	12
χ^2^		5.006		0.400		5.683	
*P*		0.024^*^		0.527		0.017^*^	

* compared with negative group, *P*< 0.05

### Diagnostic performance of HPV16/18 infection and methylation in the promoter regions of *WT1, NKX6-1* and *DBC1*

We tested and compared the sensitivity, specificity, positive predictive value and negative predictive value of gene methylation and HPV16/18 infection in normal tissue, low-grade squamous epithelial lesions (CIN1), high-grade squamous intraepithelial lesions (CIN2 and CIN3) and cervical cancer tissues. For the diagnosis of cervical cancer, methylation in the promoter region of *WT1* showed a higher specificity (94.3%), sensitivity (79.4%) and positive predictive value (94.7%) than methylation in the promoter regions of *NKX6-1* (88.7%, 73.5% and 89.3%) and *DBC1* (79.2%, 70.6% and 80.0%). For HPV16/18 infection, the specificity and sensitivity were 84.9% and 61.8%, and the positive predictive value and the negative predictive value were 84% and 63.4%. Methylation had a higher sensitivity than HPV16/18 infection. Furthermore, the specificity and sensitivity of the combined methylation analysis were 81.1 and 86.7%, respectively (Table 8).

**Table 8:** Sensitivity and PPV to detect CIN2, CIN3 or cancer, and NPV and specificity for normal or CIN1.

	Sensitivity		Specificity	Positive predictive value	Negative predictive value	
	CIN2 and CIN3/Cancer	Normal	Normal/CIN1	CIN2 and CIN3/Cancer	Normal	Normal/CIN1
HPV16/18	42/68 (61.8%)	37/43(86.1%)	45/53(84.9%)	42/50(84%)	37/71 (52.1%)	45/71(63.4%)
WT1	54/68(79.4%)	40/43(93.0%)	50/53(94.3%)	54/57(94.7%)	40/64(62.5%)	50/64(78.1%)
NKX6-1	50/68(73.5%)	38/43(88.4%)	47/53(88.7%)	50/56(89.3%)	38/65(58.5%)	47/65(72.3%)
DBC1	48/68(70.6%)	33/43(76.7%)	42/53(79.2%)	48/60(80.0%)	33/61(54.1%)	42/61(68.9%)
WT1/NKX6-1/DBC1	59/68(86.7%)	36/43(83.7%)	43/53(81.1%)	47/66(71.2%)	36/55(65.5%)	43/55(78.2%)

NPV: negative predictive value; PPV: positive predictive value.

### Gene expression

**Figure 3:**
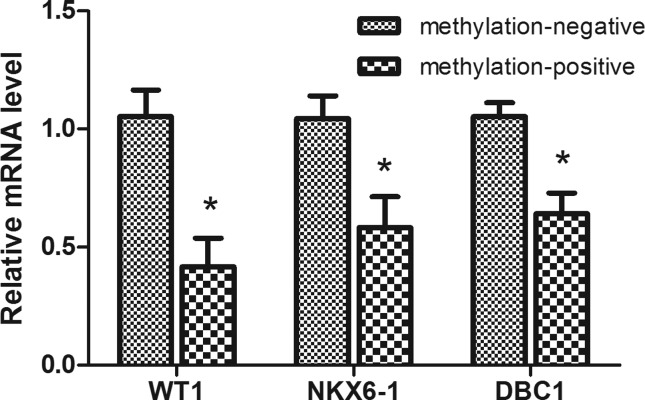
Expression of the *WT1, NKX6-1* and *DBC1* genes in 10 methylation-positive tissues and 10 methylation-negative tissues. β-actin served as internal control. Note: * statistically significant compared with methylation-negative group, *P*< 0.05.

The transcript levels of WT1, NKX6-1 and DBC1 in methylation-positive tissues were 0.416±0.387, 0.582±0.415, and 0.642±0.272, respectively. In methylation-negative tissues, the expression levels of these genes were 1.053±0.349, 1.043±0.308, and 1.052±0.187. The expression levels in methylation-positive cases were significantly lower than in the methylation-negative cases (Figure 3).

## Discussion

Cervical cancer is a preventable and treatable disease with early diagnosis and treatment. Active treatment can effectively alleviate the disease and increase the survival rate of patients. By understanding the mechanisms of cervical cancer, we hope to identify potential biomarkers for its early diagnosis.

Persistent infection with high-risk human papilloma virus (hr-HPV) is an important factor in cervical cancer incidence (Ribeiro *et al.*, 2015). However, HPV virus alone cannot cause cervical cancer. Due to individual immune defenses, most HPV infections can be removed in two years without causing any clinical symptoms and physical discomfort. The infection rate of the high-risk HPVl6 in China is 79.6% and is significantly higher than in other countries (Lo *et al.*, 2002). HPV16 has the highest infection rate, followed by HPV18, HPV58 and HPV52 (Davies *et al.*, 2001). Zuo *et al.* (2014) proposed that cervical cancer in Uygur women in Xinjiang is correlated with multiple HPV infections.

Infection of HPV16 and other HPV types accounts for 97% of the multiple infections in cervical cancer (Sohrabi *et al.*, 2017). Our results showed that HPV16 infection rates were 14.0, 36.7 and 66.7%, respectively, in the normal cervical tissues, CIN and cervical cancer tissues. Pairwise comparisons showed that the difference in HPV16 infection was statistically significant among these three groups (*P*< 0.01). The HPV18 infection rates were 0, 6.7 and 10.4% in the same groups, with no significant difference. Our results showed that HPV16/18 infection rates in the tested tissues were gradually increasing along with the degree of pathological changes (Tables 2 and 3). In the present study, we tested only HPV16 and HPV18, although there are more than 20 other types of high risk HPV.

*WT1* is a new gene with hypermethylation status in malignant tumors (Rauscher 3rd, 1993). We found that the methylation rate of the *WT1* promoter region in the analyzed tissues gradually and significantly increased along with the development of the disease. Our results indicate that methylation in the *WT1* promoter region increases significantly in cervical cancer and high-grade squamous intraepithelial lesions in comparison to normal cervical tissues of Uygur women in Xinjiang. Our results are consistent with previous findings from other regions and ethnic groups (Zhang *et al.*, 2012). The methylation rate in the promoter region of *NKX6-1* increased from the normal cervical tissues to CIN and cervical cancer tissues, which is consistent with other studies (Lai *et al.*, 2008). The methylation rate of the promoter of *DBC1* also increased from normal cervical tissues to CIN tissues and the cervical cancer tissues. However, the methylation rates of these genes in cervical cancer tissues were not significantly correlated with age and FIGO stages.

Schlecht *et al.* (2015) showed that abnormal methylation was associated with HPV infection. Henken *et al.* (2007) proposed that HPV infection could cause epigenetic reconstruction of a host cell in the process of malignant transformation, resulting in HPV phenotype in cervical cancer tissues. Leonard *et al.* (2012) proposed that HPV could also induce changes in DNA methylation transferase activity. Whether methylation in the promoter regions of *WT1, NKX6-1* and *DBC1* is associated with HPV infection is unclear. We found that methylation in the promoter regions of *WT1* and *DBC1* genes is associated with HPV16/18 infection in cervical cancer tissues of Uygur women in Xinjiang. However, methylation in the promoter region of *NKX6-1* gene was not associated with HPV 16/18 infection in cervical cancer tissues. Thus, the methylation of *NKX6-1* and HPV16/18 infection appear to be independent factors in the development of cervical cancer. For the diagnosis of cervical cancer, we tested and compared the sensitivity, specificity, positive predictive value and negative predictive value of gene methylation and HPV16/18 infection. Sensitivity is the number of positive HPV16 or HPV18 divided by the number of CIN2, CIN3 and tumor samples (42/68). Specificity is the number of negative HPV16 and HPV18 divided by the number of normal samples (37/43). Gene methylation detection was also calculated according to this method. Our results showed that methylation in the promoter regions of *WT1* and *NKX6-1* had higher sensitivity, specificity, positive predictive value and negative predictive value than HPV16/18 infection. In addition, the combined methylation analysis of *WT1, NKX6-1* and *DBC1* had a higher sensitivity than individual genes. Considering that screening for gene methylation of cervical lesions is more reliable than detection of HPV16/18 infection, the probability of misdiagnosis by gene methylation is greatly reduced. Therefore, gene methylation provides a more reliable molecular marker for the diagnosis of cervical cancer of Uygur women.

The expression of *WT1*, *NKX6-1* and *DBC1* genes in methylation-positive cervical cancer tissues was significantly lower than in methylation-negative normal cervical tissues. Thus, gene methylation may lead to gene inactivation and play a role in the genesis and development of cervical cancer.

In conclusion, Uygur women in Xinjiang are a high-risk population for cervical cancer. It is important to understand cervical cancer pathogenesis and develop suitable diagnosis and treatment strategies. Cytological diagnosis of cervical cancer usually requires specimens collected by surgery or biopsy. However, the sensitivity of cytological diagnosis is low (Nanda *et al.*, 2000) and there are different standards (Yang *et al.*, 2009). Gene methylation is a convenient marker for early diagnosis and screening of tumors. We showed in this study that methylation rate in the promoter regions of the *WT1, NKX6-1* and *DBC1* genes were higher in cancer than in normal tissues and the expression of these genes was lower in cervical cancer of Uygur women than in the methylation-negative normal cervical group. These three genes may be suitable molecular markers for diagnosis of cervical cancer.
